# ASO Author Reflections: Surgical Management of Urachal Cancer in Germany: A Nationwide Analysis from 2006 to 2022

**DOI:** 10.1245/s10434-026-19977-4

**Published:** 2026-06-15

**Authors:** Luka Flegar, Nicole Eisenmenger, Philipp Reimold, Rainer Koch, Johannes Huber, Christer Groeben

**Affiliations:** 1 Department of Urology, University of Heidelberg, Heidelberg, Germany; 2 Reimbursement Institute, Hürth, Germany

## Past

Surgical management of urachal cancer typically involves partial or radical cystectomy with en bloc removal of the urachus and umbilicus.^1^ Guidelines generally favour partial cystectomy to preserve bladder function and quality of life. Advances such as minimally invasive approaches, improved imaging and centralized care may be reshaping practice patterns; however, robust population-based evaluations of these shifts remain limited.^2^ To address this gap, this study examines contemporary treatment trends for urachal cancer over a 16-year period, assessing adoption of minimally invasive techniques and alignment with guideline recommendations.

## Present

By analysing the nationwide German hospital billing database (Destatis) and German quality reports a total of 2593 cases were included. The share of partial cystectomy for treatment of urachal cancer increased significantly to 74% in 2022. Driven by advancements in perioperative care and recovery protocols, the average hospital stay after a partial cystectomy dropped from 14 days in 2006 to just 9 days in 2022. Robotic-assisted partial cystectomy further accelerated this recovery, resulting in an even shorter length of stay. Continuous bladder irrigation and vacuum-assisted closure (VAC) placement were the most common procedures associated with postoperative complications following urachal cancer treatment. Furthermore, the present study highlights potential gaps in Germany’s nationwide care coverage.^3^

## Future

Moving forward, the rarity and aggressive nature of urachal cancer necessitate national and international collaboration to establish optimal treatment strategies. Furthermore, multicentre studies are required to investigate whether the observed shift toward partial cystectomy compromises or maintains oncological efficacy.
